# C2 and Greater Occipital Nerve: The Anatomic and Functional Implications in Spinal Surgery

**DOI:** 10.7759/cureus.1074

**Published:** 2017-03-03

**Authors:** M. Burhan Janjua, Peter L Zhou, Jeffrey P Greenfield, Ali A Baaj, Anthony Frempong-Boadu

**Affiliations:** 1 Neurosurgery/Spine Surgery, New York University Langone Medical Center; 2 Hospital for Joint Diseases, New York University Langone Medical Center; 3 Neurological Surgery, New York-Presbyterian/Weill Cornell Medical College

**Keywords:** c2 neurectomy, occipital neuralgia, lateral mass screws, greater occipital nerve, c1-c2, c1-c2 facet joint

## Abstract

**Introduction:**

Posterior C1-C2 fusion is a highly successful treatment for atlantoaxial instability and other pathologies of the cervical spine, with fusion rates approaching 95%-100%. However, poor visualization of the lateral masses of C1 secondary to the course of the C2 nerve root along with blood loss from the venous plexus and compression of the C2 nerve from lateral mass screws are technical obstacles that can arise during surgery. Thus, sacrifice of the C2 nerve root has long since been debated in fusions involving the C1 and C2 vertebral bodies.

**Methods:**

Cadaveric dissections on four adult specimens were performed. Both intradural and extradural courses of C2 were studied in detail. The tentative site of C2 nerve root compression during placement of C1 lateral mass screws was studied in detail. Both the indication as well as the ease of C2 neurectomy were studied in relation to postoperative compression and entrapment.

**Results:**

Four-six dorsal rootlets of C2 nerve were observed while studying the intradural course. The extradural course was studied with respect to the lateral mass of C1. The greater occipital nerve (GON) course was fairly consistent in all specimens. Transection of C2 around its ganglion would allow for proper C1 lateral mass screw placement as the course of C2 nerve interferes with proper placement of instrumentation.

**Conclusion:**

C2 nerve root transection is associated with occipital numbness but this often has no effect on health-related quality of life (HRQOL). The C2 nerve root preservation is often associated with entrapment neuropathy or occipital neuralgia, which greatly affects HRQOL. The C2 nerve root transection helps in better visualization, aids in optimal placement of C1 lateral mass screws, minimizes estimated blood loss and improves surgical outcome with successful fusion.

## Introduction

Posterior C1-C2 (atlantoaxial) instrumentation and fusion are a surgical procedure employed to treat pathologies of axial cervical spine. There are various etiologies, which include degenerative and metabolic bone or ligamentous abnormalities, infectious or inflammatory joint diseases, congenital anomalies (os odontoideum and odontoid agenesis), trauma with destabilizing fractures, tumor, and iatrogenic causes from previous decompression surgeries. Atlantoaxial instability (AAI) can be asymptomatic in its mildest form, however, when symptomatic warrants C1-C2 fixation. 

C1-C2 instrumented fusion can provide a reasonable relief from symptoms but it comes at the expense of loss of some rotational mobility of the cervical spine. Usually, surgery is limited by the complicated anatomy and the intricate course of the neurovascular structures in narrow and confined spaces. Multiple surgical techniques have evolved over the last 70 years to address AAI. In 1979, Magerl and Jeanneret described transarticular screw fixation. Later on, Goel’s technique using C1 lateral mass screws and C2 pedicle screws with plates for posterior C1-C2 fusion has traditionally been considered a gold standard [1]. The technique was later revised by Harms, et al. by utilizing rods with C2 pars, and C1 lateral mass screw construct, now considered by many to be more feasible and favored technique [2]. As surgical advancements are made, the use of C2 nerve transection arose from cumbersome dissections, profuse bleeding from perivertebral venous plexus in the lateral gutters, anomalous course of neurovascular structures, poor visibility in decortication around the facet joint and foremost the deployment of C1 lateral mass screws.

The C2 nerve root occupies the dorsal aspect of the C1-C2 facet joint and obscures important landmarks for correct screw placement. Moreover, the implanted hardware can cause impingement or compression of the nerve root, which can lead to the development of neuropathic pain in the cutaneous distribution of C2 nerve (occipital neuralgia) [3-5]. Thus, preserving or sacrificing the nerve root has been a controversial issue where the decision is based on surgical experience and personal preference to enhance overall functional outcome.

This cadaveric study aims to delineate important anatomical landmarks for proper C1 screw placement and avoidance of postoperative occipital neuralgia. Furthermore, we aim to highlight the importance of anatomic landmarks of the posterior C1-C2 interspace while considering posterior instrumentation to minimize postoperative neuropathic pain when preserving C2. This study also details the microsurgical anatomy of the C2 nerve, its intradural and extradural course and the feasibility of either preserving or sacrificing C2 during posterior C1-C2 instrumentation and fusion.

## Materials and methods

Four formalin-fixed adult cadaveric specimens with well-preserved cervical spines up to the C4 spinous process were selected. Each specimen was placed in a three-point pin fixation device (Mayfield) in a prone position. The cervical spine was exposed from occiput to C3 in order to study the vital neuromuscular structures in the posterior C1-C2 disc space. Standard midline suboccipital approach was utilized for all specimens. In total, eight sides were dissected. The whole C2 nerve course starting from intradural dorsal rootlets to the extradural portion up to the terminal skin innervation of the vertex was studied bilaterally in all specimens. All microdissections of the C1-C2 disc space were performed under the surgical microscope.

Cadaveric Dissection Details and Course of C2 Nerve

**Figure 1:**
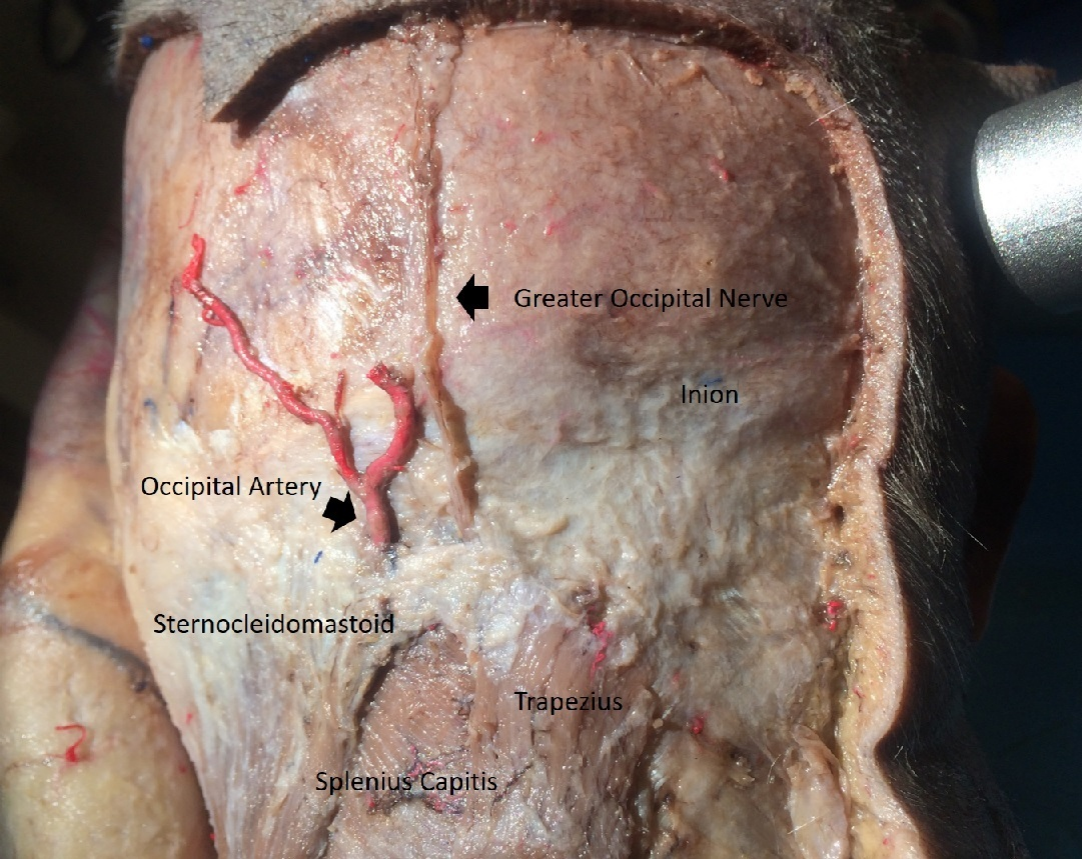
Suboccipital region depicting GON

A midline incision was performed up to the spinous process of C4. The skin and subcutaneous tissue were then carefully dissected. A meticulous subdermal dissection was carried out to expose the fascia of the trapezius muscle. Then, in a stepwise fashion, fascia was dissected to yield the opening of the greater occipital nerve (GON), a medial branch of the dorsal ramus of C2. The point of exit of the GON out of the aponeurosis of the trapezius muscle just lateral to the inion was observed in all specimens. This course was relatively consistent in all cadaveric specimens (Figure 1).

**Figure 2:**
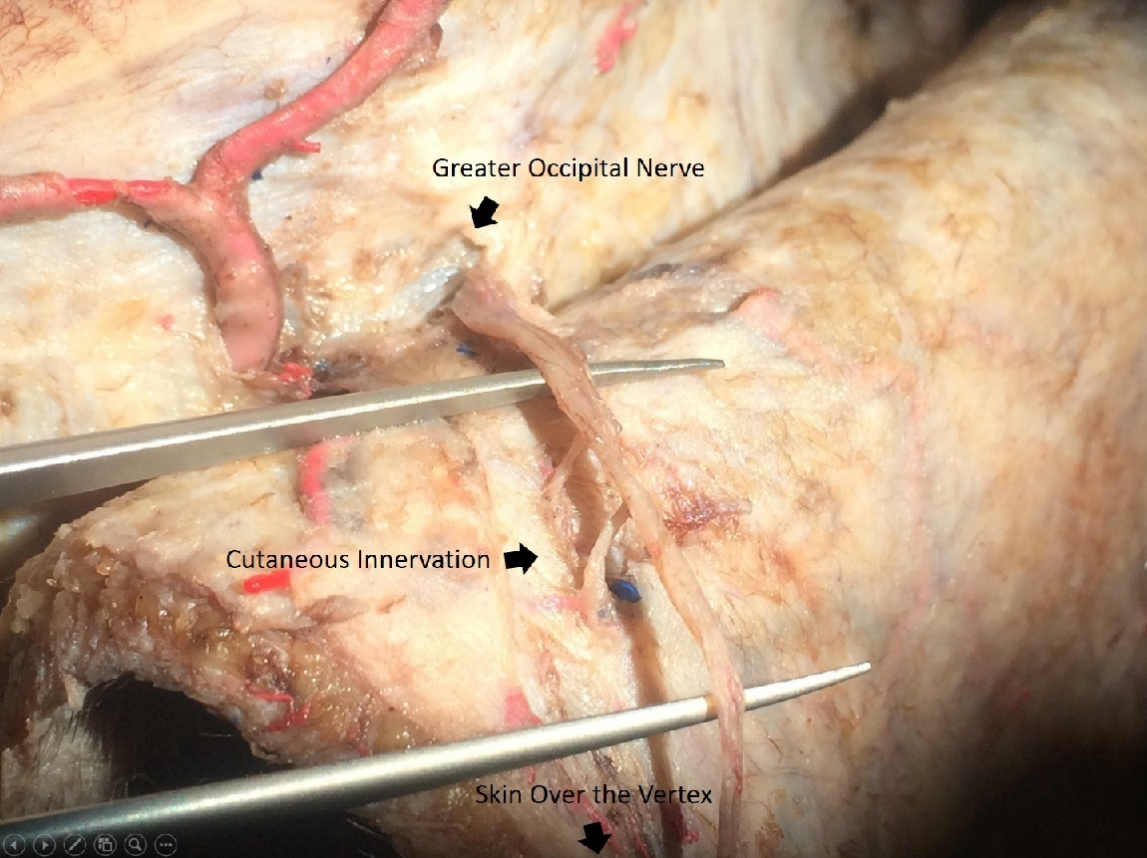
Cutaneous innervation over vertex The course of GON after exiting after the fascia lateral to the inion

During its course between trapezius and semispinalis capitis, the GON was noticed to receive a medial branch from C3. The skin incision was advanced further upwards to expose the terminal cutaneous innervation of C2 nerve up to the vertex (Figure 2).

**Figure 3:**
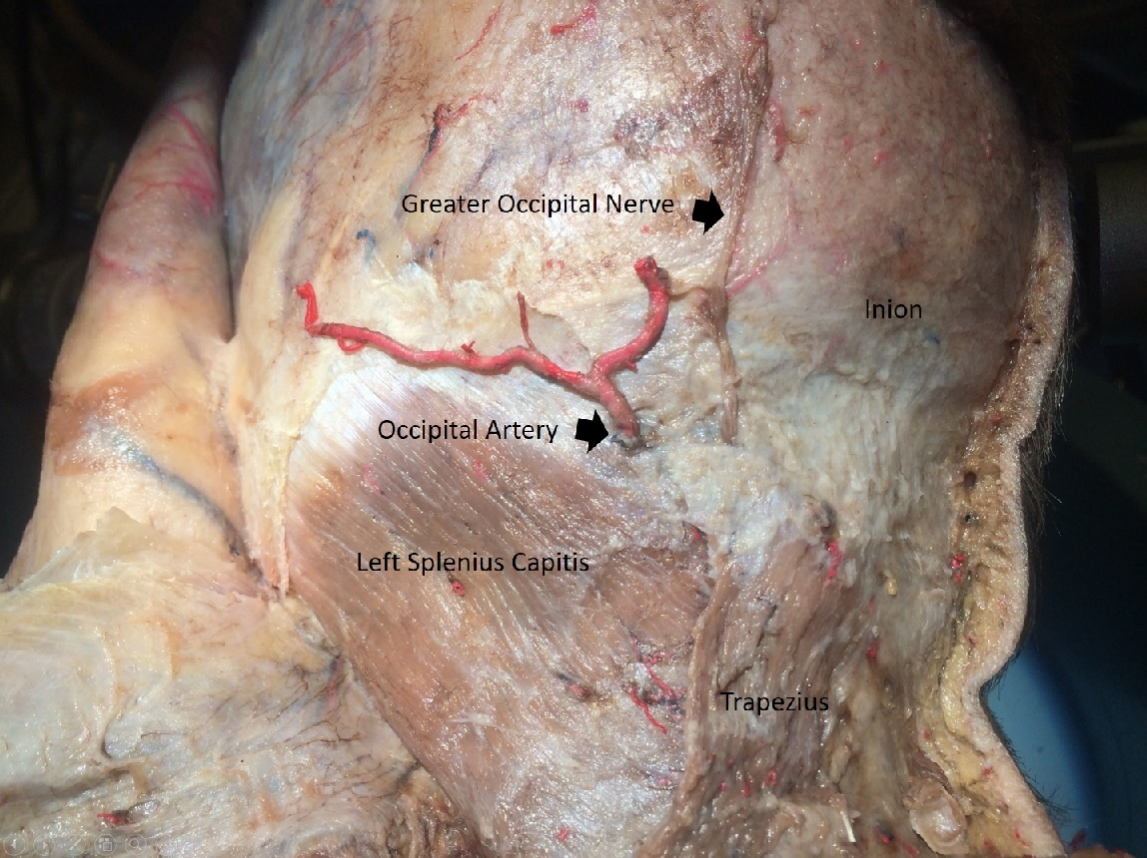
Trapezius split open to expose splenius capitis

Then, the dissection was extended laterally to expose the fascia of the splenius capitis muscle. Afterward, the midline incision was extended subfascially through the trapezius muscle (Figure 3).

**Figure 4:**
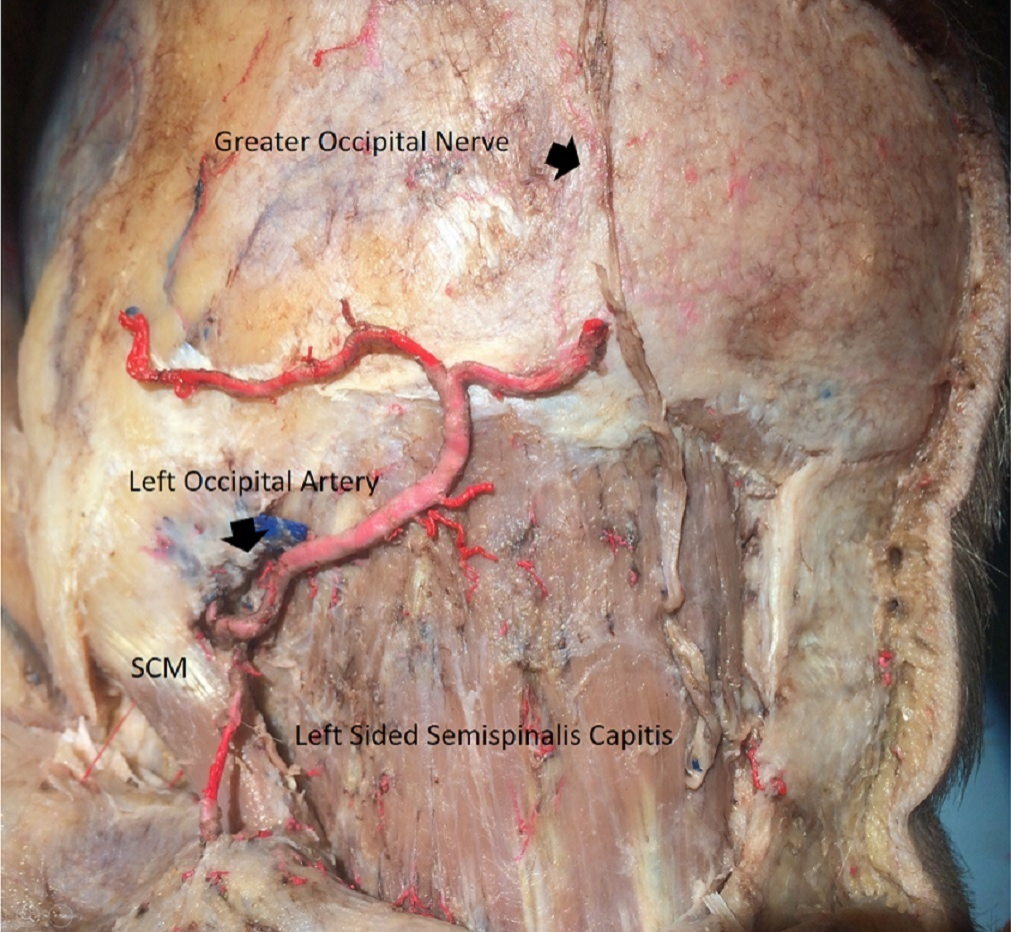
Splenius capitis dissected out Splenius capitis was dissected out to expose the semispinalis capitis

Followed by a lateral retraction to expose the semispinalis capitis muscle (Figure 4).

**Figure 5:**
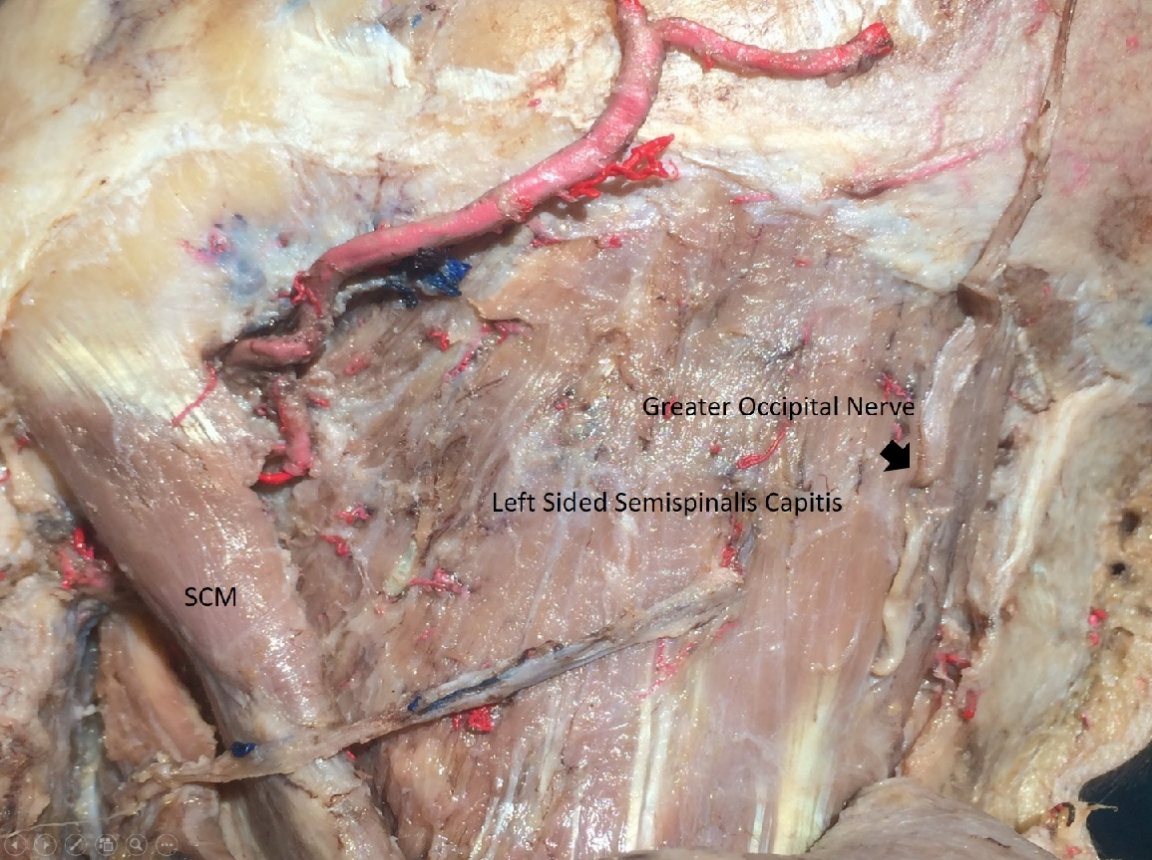
The GON exiting semispinalis capitis

**Figure 6:**
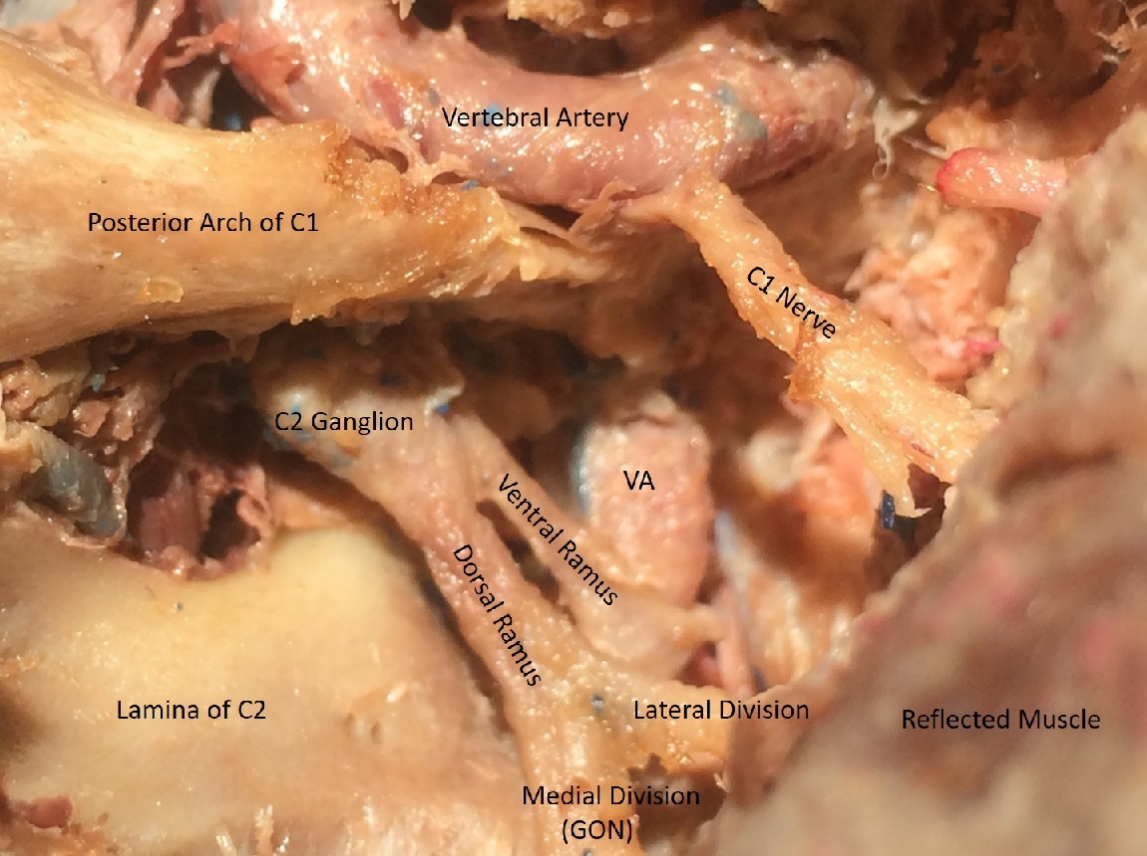
Posterior C1-C2 interspace C2 ganglion and post-ganglionic segments with ventral and dorsal rami

The GON could be identified exiting the muscle (Figure 5),

and was then traced back to the inferior border of the inferior oblique muscle and to the posterior C1-C2 interspace (Figure 6). 

**Figure 7:**
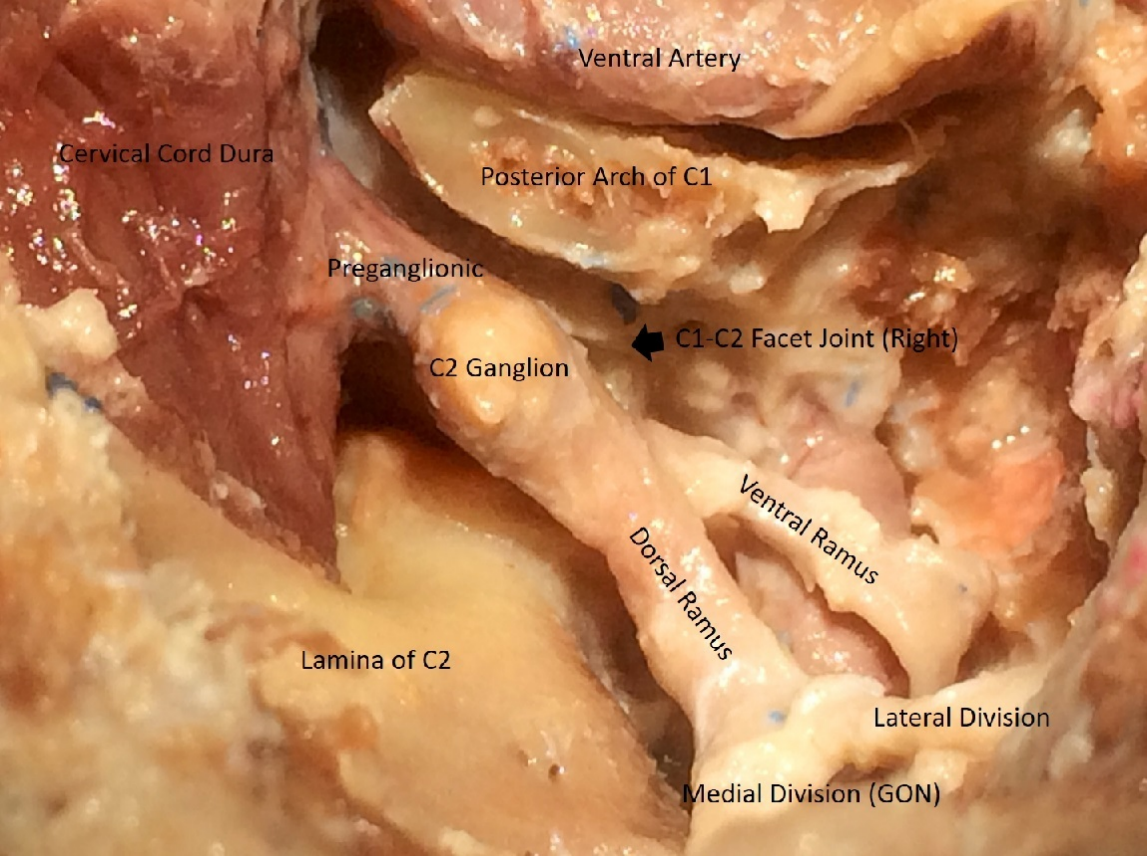
Pre and post-ganglionic segments of C2 behind the facet joint

The dissection was then carried down to the C2 spinous process to expose the muscles attached to the spinous process. Subperiosteal dissection of the C2 nerves was carried out over the lamina bilaterally up to the C1-C2 facet joint (Figure 7).

**Figure 8:**
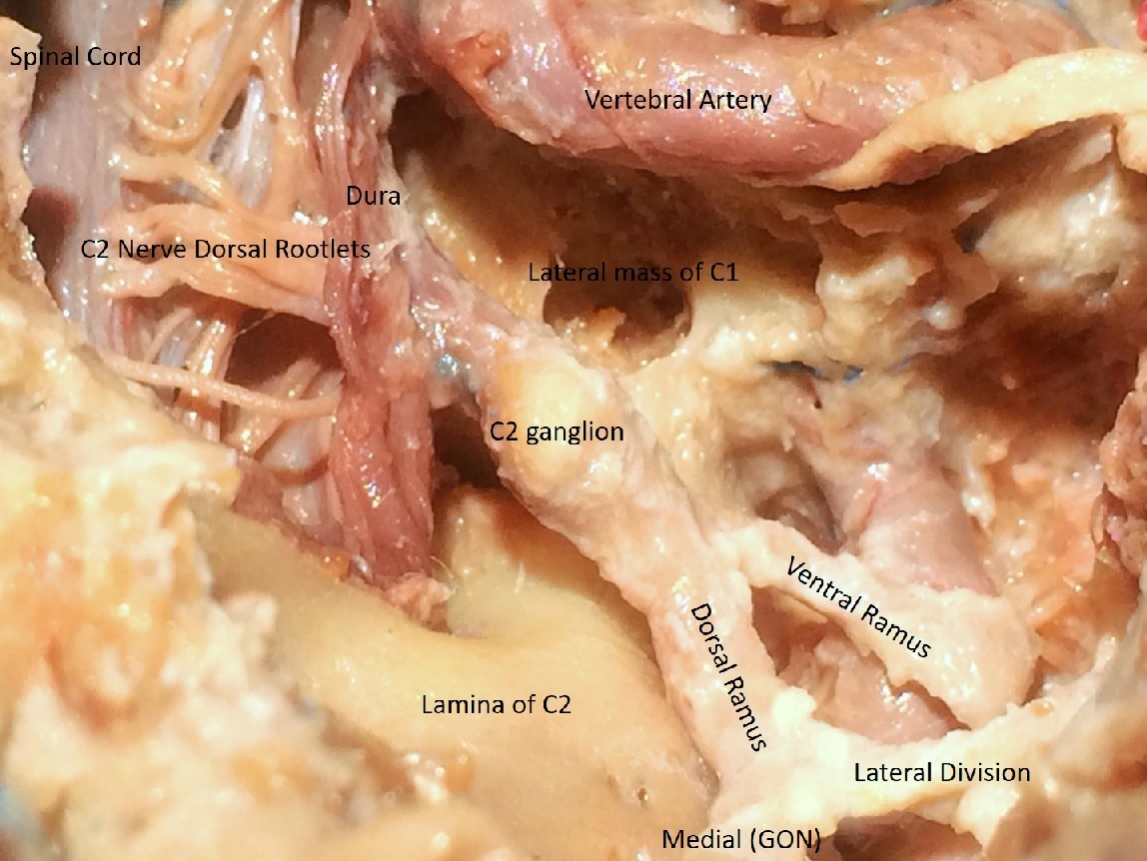
Intra and extradural course of C2 The extradural course of C2, along with its rami and divisions, in the posterior C1-C2 disc space

A C1 laminectomy, utilizing a 4mm cutting bar was subsequently performed to expose the spinal dura. The drilling was extended laterally while carefully preserving the vertebral artery running through the sulcus arteriosus. The vertebral artery was completely skeletonized up to the foramen transversarium. This led us to expose the C1 nerve root exiting under the vertebral artery. The C1 nerve, also known as the suboccipital nerve was observed leaving the vertebral canal between the occipital bone and the C1 vertebra. The dorsal ramus was found to be larger than the ventral ramus and coursed between the posterior arch of the C1 and the vertebral artery to reach the suboccipital triangle (formed by the rectus capitis posterior major, superior and inferior oblique muscles) (Figure 7). A durotomy was then performed utilizing size 15 blade in a standard fashion. The edges of the dura were cut laterally to expose the lateral extent of the cervical spinal cord. Four-six dorsal rootlets of C2 were identified exiting from the dorsal root entry zone of the dorsal segment of the spinal cord (Figure 8). Ventral rootlets were not dissected and limited by the suboccipital approach only. 

The C2 nerve root along with the C2 ganglion were identified. The number of C2 nerve rootlets, the proximal and distal location of C2 ganglion, its shape, and location relative to the posterior aspect of the lateral mass of C1 vertebra were closely observed (Figure 8).

## Results

The GON and its course were fairly consistent in all specimens from inferior border of the inferior oblique muscle and up to the posterior C1-C2 disc space. However, the diameter of the preganglionic, ganglionic or postganglionic segments of C2 was found to be variable (not measured) in all specimens. After durotomy, four-six dorsal rootlets of C2 were observed in all specimens. The C2 nerve was observed to emerge from the spinal dura between the posterior arch of the C1 and the lamina of the C2 vertebra and continued as the C2 ganglion extradurally, either just medial or dorsal to the lateral mass of C1, and the C1-C2 facet joint and medial to the vertical segment of the vertebral artery. Distal to the ganglion, the C2 nerve divided into a larger dorsal and a smaller ventral ramus. The ventral ramus passed behind the vertical segment of the vertebral artery and traversed forward and laterally. After supplying the inferior oblique muscle, the dorsal ramus was noticed to divide into a small lateral and a large medial division. The medial division continues as GON. The lateral branch was dissected only leaving the posterior interspace. The GON was observed to climb between semispinalis capitis and trapezius muscle and exiting lateral to the inion to innervate the skin up to vertex. Special attention was focused on observing the relative position of the C2 ganglion with respect to the lateral mass of the C1 vertebra. Interestingly, in all specimens it was noticed that the ganglion rests right behind the lateral mass of C1 vertebra and significantly hinders the safe deployment of C1 lateral mass screws.

## Discussion

In this study, we delineated the anatomical borders in the posterior aspect of the atlantoaxial region and identified the location of both the C2 nerve root and its ganglion in relation to the surrounding structures. We recognized that the nerve hindered the proper placement of C1 lateral mass screws and access to C1-C2 facet joints for decortication and/or placement of cages. This may contribute to post-operative complications such as occipital neuralgia and suboptimal fusion.

Although not considered as a true objective measure of functional outcome, some patients have complained of postoperative occipital numbness [6]. Fortunately, these patients are often times never uncomfortable enough to call it a true neuropathic pain. Aryan, et al. followed 102 patients who had C2 neurectomies as part of posterior C1-2 instrumented fusions. Only one patient complained of neuropathic pain in the sensory distribution without any reports of hypesthesia [7]. Another study found similar results in 20 patients with none of the patients developing a true neuropathic pain postoperatively, only five patients developing occipital anesthesia and another five developing hypesthesia. Only one patient with sensory disturbance reported a negative effect on health-related quality of life (HRQOL) due to daily occurrence [8]. All other patients reported much less frequent occurrences at weekly and monthly intervals. Dewan, et al. prospectively followed 28 patients who underwent atlantoaxial fusion with or without C2 neurectomy (eight transections, 20 preservations), and found that while 50% of patients who underwent transection complained of occipital numbness, none reported a negative effect on HRQOL. On the other hand, 35% of patients who had their C2 nerves preserved complained of occipital neuralgia with all of them reporting the symptom to be bothersome and having significant effect on HRQOL [9]. Similar results were observed in other studies where patients reported relief of preoperative neck pain or radicular pain at the expense of minimal numbness in the C2 distribution with excellent results in fusion [6-7,10,12]

Not all literature is in agreement with the relative benign prognosis of undergoing a C2 neurectomy. Yeom, et al. compared 24 patients who had bilateral C2 transections with 41 patients without neurectomies. Patients who had neurectomies were found to have a greater percentage in incidences of postoperative neuralgic pain. Interestingly, they also found that the intensity of pain was much greater postoperatively in the patients who underwent transections. However, it should be noted that in their cohort, the ganglion was not completely dissected prior to transection which leads to the nerve root being transected through the ganglion itself rather than proximal to it [13]. Transection proximal to the ganglion is preferred as the cell bodies, are located within the ganglion and may be a cause of occipital pain if, damaged or compressed [14]. Isolated C2 nerve root pain is usually not treated with C2 neurectomy, as management is focused on treating the primary cause of pain. However, patients with C2 nerve root pain resulting from aberrant nerve root regeneration subsequent to incomplete destruction of the dorsal root ganglion respond well to C2 neurectomy and/or ganglionectomy [15-16].

A meta-analysis performed by Elliot, et al. found not only improved rates of occipital neuralgia in patients after C1-C2 fixation with C2 neurectomies, but also found that sacrifice of C2 was associated with reduced estimated blood loss (EBL), estimated operative time, and neuropathic pain. Also, there was no effect on the rates of fusion [17]. Similar results were found in other studies [9,11]. From our experience, a meticulous subperiosteal dissection (rostral to caudal) of the C1-C2 joint is a crucial step in posterior instrumentation. Coagulation of the venous plexus around the C2 nerve root proves important for the complete visualization of the C1 lateral masses and deployment of screws [8,16,18]. The C2 nerve, due to its positioning relative to the C1 lateral masses, often necessitates increased operative time in order to dissect out the surrounding structures, which can lead to increased EBL. Furthermore, C2 neurectomies can aid in visualization and feasibility in cases of C1 posterior arch nonunion or fractures as well as avoid cardiovascular instability in bleeding diathesis, aid in anterolateral access to the cervical segment of the spinal cord, and optimize decortication of the C1-C2 facet joint for successful fusion [17].

Wide exposure of the lateral mass of C1 can also be obtained by drilling the inferior half of the C1 lamina off to avoid unnecessary pressure on the C2 nerve root and ganglion. This step can help preserve the C2 nerve root but places the vertebral artery in jeopardy instead [1,8,18-19]. In another meta-analysis, it was found that atlantoaxial fusions accompanied by C2 transections were less likely to lead to vertebral artery injury as compared to C2 preservation. Furthermore, the incidences of both clinically significant and radiographic screw malpositions were much less frequent when the C2 nerve was sacrificed. Similar to previous reports mentioned, the incidence of postoperative occipital numbness was much higher in patients who had neurectomies but the rate of neuralgia was much less [20].

Through our cadaveric dissections, we highlighted the close proximity of neurovascular structures in the confined posterior C1-C2 interspace. We also highlighted that the C2 nerve is superficial, fixed in position, traverses a confined space and is also in close proximity to the C1-C2 facet joint, which predisposes it to compression or neuropathy in subluxation and facet arthropathies. Although the bony anatomy is fairly consistent, the relative diameter and position (measurements not performed) of the C2 nerve, the length of the preganglionic segment, postganglionic segment, the diameter of the C2 ganglion, and its position relative to the C1-C2 facet joint were the important parameters. Many spine surgeons advocate for complete excision of the C2 nerve root plus ganglion but care has to be taken to avoid excising the ganglion too proximal from the dural ring to avoid intractable postoperative cerebrospinal fluid (CSF) leak [10,14]. C2 neurotomy and/or ganglionectomy should be a safe and watertight procedure. Double ligation of the stump is a less favourable option to prevent the efflux of CSF postoperatively [21]. Apart from hypoesthesia and CSF leakage, there have also been reports of alopecia from denervation after transection of the C2 nerve [14].

In our specimens, we recommend the benefit of transection of the C2 nerve root in order to identify clear bony landmarks for easy C1 screw deployment and decortication for optimal fusion. As no two patients are identical, a spine surgeon must have a full appreciation of each patient’s anatomy. Understanding of the anatomy of the C1-C2 region allows for safe deployment of instrumentation with minimal risk of cortical breakage or injury to the neurovascular structures. While there are guidelines for the proper placement of posterior C1-C2 pars screws, adherence to such algorithmic details is limited when taking into account individual anatomic variations. Preserving the nerve root in an attempt to maintain neural integrity can jeopardize patient outcomes due to the complaints of postoperative occipital neuralgia.

There are limitations to this study that are appreciated. The sample size of specimens was limited. Furthermore, cadaveric specimens may not fully represent what is seen on the operating table. 

## Conclusions

The knowledge and understanding of the intricate microsurgical anatomy of the C2 nerve root are essential for proper placement of C1 lateral mass screws. The course of C2 nerve around the facet joint necessitates its removal in procedures requiring C1 lateral mass screw insertion. C2 neurectomy offers a reasonably safe dissection zone to avoid inadvertent vertebral artery injury while facilitating access to the C1-C2 joint for preparation, cage placement, and fusion. While occipital numbness may develop, the more severe complication of postoperative occipital neuralgia is avoided.
